# Homologies between SARS-CoV-2 and allergen proteins may direct T cell-mediated heterologous immune responses

**DOI:** 10.21203/rs.3.rs-86873/v1

**Published:** 2020-10-06

**Authors:** Kathrin Balz, Meng Chen, Abhinav Kaushik, Franz Cemic, Vanessa Heger, Harald Renz, Kari Nadeau, Chrysanthi Skevaki

**Affiliations:** 1 Institute of Laboratory Medicine, Universities of Giessen and Marburg Lung Center (UGMLC), Philipps University Marburg, German Center for Lung Research (DZL), Marburg, Germany; 2 Sean N. Parker Center for Allergy and Asthma Research at Stanford University and Division of Pulmonary, Allergy & Critical Care Medicine, Stanford, CA, USA.; 3 TH Mittelhessen, Department of Computer Science, University of Applied Sciences Gießen, Hessen, Deutschland

## Abstract

The outbreak of the new Severe Acute Respiratory Syndrome Coronavirus-2 (SARS-CoV-2) is a public health emergency. Asthma does not represent a risk factor for COVID-19 in several published cohorts. We hypothesized that the SARS-CoV-2 proteome contains T cell epitopes, which are potentially cross-reactive to allergen epitopes. We aimed at identifying homologous peptide sequences by means of two distinct complementary bioinformatics approaches. Pipeline 1 included prediction of MHC Class I and Class II epitopes contained in the SARS-CoV-2 proteome and allergens along with alignment and elaborate ranking approaches. Pipeline 2 involved alignment of SARS-CoV-2 overlapping peptides with known allergen-derived T cell epitopes. Our results indicate a large number of MHC Class I epitope pairs including known as well as *de novo* predicted allergen T cell epitopes with high probability for cross-reactivity. Allergen sources, such as *Aspergillus fumigatus*, *Phleum pratense* and *Dermatophagoides* species are of particular interest due to their association with multiple cross-reactive candidate peptides, independently of the applied bioinformatic approach. In contrast, peptides derived from food allergens, as well as MHC class II epitopes did not achieve high *in silico* ranking and were therefore not further investigated. Our findings warrant further experimental confirmation along with examination of the functional importance of such cross-reactive responses.

## Introduction

The World Health Organization (WHO) has declared the outbreak of the new Severe Acute Respiratory Syndrome Coronavirus-2 (SARS-CoV-2, ssRNA virus, associated with COVID-19) as a public health emergency. As per the WHO report of 20 September 2020, more than 30 million cases and over 950000 deaths have been reported worldwide.^1^ Human coronaviruses are positive-sense single-stranded RNA (+ssRNA) viruses, with SARS-CoV-2 and SARS-CoV belonging to the B-lineage of the Betacoronavirus genera and MERS-CoV to the C-lineage of the same genera.^2,3^ The clinical features in patients affected with these respiratory viruses ranges from asymptomatic carriers to severe respiratory illness with pneumonia and acute respiratory distress syndrome (ARDS). In addition, a number of interesting vascular and inflammatory presentations have been noted, including a multisystem inflammatory syndrome in children.

We have previously reported on heterologous immune responses induced by influenza, another respiratory RNA virus, against allergens, which mediated protection from experimental allergic asthma.^4^ Indeed, virus-induced T cell mediated heterologous immunity has been widely described in a variety of settings, which can confer protection or drive immunopathology against other antigens.^5,6^ Given that the host immune response to SARS-CoV-2 and associated disease course can be so varied from patient to patient, this spectrum of presentations raises the question of what drives the differential host immune response. There is still little known about asthma phenotypes and severity of COVID-19. In general, asthma has not been shown to be a risk factor for COVID-19 in several published cohorts.^7,8^ However, recent studies from the UK and the USA indicated higher numbers of asthmatics in COVID-19 patients.^9^

Interestingly, the UK Biobank recently reported that non-allergic patients had a higher risk of severe COVID-19, compared to patients with allergic asthma^10^. Moreover, increased numbers of activated T cells were found among asthmatic COVID-19 patients who showed a less severe disease, suggesting that activated T cells have a positive impact on severity of SARS-CoV-2 infection^11^. These preliminary clinical observations along with our prior experimental evidence involving RNA viruses led us hypothesize that SARS-CoV-2 may share a degree of protein sequence homology to allergens, which may lead to the generation of cross-reactive T cell epitopes. Pre-existing T cells specific for such cross-reactive allergen-derived epitopes may have an impact on COVID-19 outcome via aberrant cytokine responses to the virus peptides. Indeed, these cytokines could prevent an overshooting T1 inflammatory reaction, both locally (as in the case of preexisting pulmonary CD4^+^ T cells specific to inhalant allergens) and/or systemically. Therefore, we sought to predict potentially cross-reactive allergen- and SARS-CoV-2-derived MHC Class I and Class II T cell epitopes, which can be presented by the most prevalent HLA alleles.

## Methods and Results

**Figure 1:**
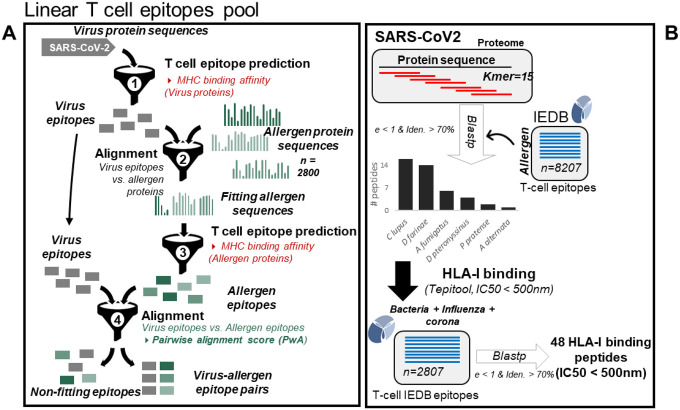
Schematic overview of the bioinformatics approaches A: Pipeline 1; SARS-CoV-2 proteins were aligned against >2500 allergen protein sequences (see methods) and MHC class I-and II- restricted potentially cross-reactive T cell epitope pairs were identified for the most frequent human HLA alleles. B: Pipeline 2; In an independent framework, we performed the comparative analysis of sequential *kmers* from SARS-CoV-2 protein sequences with known IEDB allergen peptides to predict the cross-reactive viral peptide pool.

In order to examine our working hypothesis, we applied two distinct independent, complementary and systematic bioinformatics approaches (Figure 1): a) Pipeline 1-prediction of MHC Class I and Class II epitopes contained in the SARS-CoV-2 proteome and a comprehensive set of allergen protein sequences combined with alignment strategies and ranking of results based on clinical and sequence conservation criteria and b) Pipeline 2- alignment of SARS-CoV-2 overlapping peptides with known allergen-derived T cell epitopes.^12^

### Pipeline 1

More than >2500 allergen protein sequences were downloaded (dates of access 10.09.2017) from Allergome^13–16^ (Supplementary Table S1), and protein sequences for SARS-CoV-2 from UniProt^17^ (Supplementary Table S2). Viral T cell epitope prediction was performed using smm^18^, ann^19^ and consensus^20^ for MHC Class I (IC50 threshold <=5000), and netMHCII^21^ for MHC Class II (affinity score threshold for strong binders: 0.500; for weak binders: 2.000) (Supplementary Table S3). Epitopes predicted by all methods were aligned against all allergen proteins with NCBI protein blast platform^22^. Allergen proteins associated with an alignment e-value <10 were further processed for T cell epitope prediction using netMHC^23^ and netMHCpan^24^ for MHC Class I, and netMHCII and netMHCIIpan^25^ for MHC Class II prediction (affinity score threshold for strong binders: 0.500; for weak binders: 2.000). Viral and allergen epitopes were pairwise aligned with Biopython module pairwise 2^26^ and for pairs with a score > 8, a final pair combined score (pcs) was calculated (Supplementary Methods). Duplicates among the resulting candidate epitope pairs were removed before further processing. Therefore, possible sequence repetition due to isoforms and isoallergens (Supplementary Table S1) do not influence further analyses. In total, we obtained more than 5000 candidate pairs for each, MHC Class I and Class II. The top 30 candidate epitope pairs, as per pair combined score, are listed for aero- and food allergens, MHC Class I and Class II presentation background in Supplementary Tables S4–7, respectively. The top 30 MHC Class II restricted predicted virus-allergen pairs achieved relatively low pcs (24–657) as compared to Class I epitope pairs (1036–10816). Among our top 30 MHC Class I potentially cross-reactive allergen derived epitopes, we identified more than 20 distinct protein families (Allfam database). In addition to MHC binding affinity and homology between peptide sequences, also other factors (e.g. conservation, association with clinical reactions) are important for the clinical relevance of peptides predicted to be cross-reactive at the T cell level. In order to capture this information level in our ranking, all allergen peptides and associated sources listed among the top 30 candidate epitope pairs were evaluated further with a scoring system (Supplementary Fig. S1 and Supplementary Methods). We found that the top 5 Class I aeroallergens were on average associated with higher pcs as compared to the top 5 potentially cross-reactive food allergens (Table 1 for MHC Class I and Table 2 for MHC Class II peptide pairs).

### Pipeline 2

We obtained all known allergen-derived linear T cell epitope peptides from the IEDB, containing peptides known to bind MHC molecules with at least one published experimental evidence (e.g. based on the results of a T cell assay) (Supplementary Table S8). A total of 8,207 antigenic peptides from 142 antigens were selected for evaluation, among which, peptides with ambiguous amino acids (e.g. with unknown amino acid ‘X’ or any special character) were removed from the subsequent analysis. Therefore, all included peptides could be defined in full. Next, SARS-CoV-2 protein sequences were analyzed for the potential antigenic regions by splitting each of the sequence into sequential *k-mers* (length=15), and homology with allergen antigenic peptides was then profiled. Within a given threshold range, we found 43 unique SARS-CoV-2 peptides that belong to replicase poly protein and spike glycoprotein (Supplementary Table S9). These peptides demonstrate homology with antigenic peptides of 6 different allergens, all of which are known to be respiratory allergens (e.g. aeroallergens; Figure 1). However, despite the homology, it is likely that some of the peptides may not have strong MHC Class I binding affinity, and thus be less likely to be presented as antigens by HLA molecules. Therefore, we assessed the binding affinity of these peptides with human MHC Class I molecules, across a broad range of alleles that are known to bind viral proteins (52 most common HLA-A and HLA-B alleles). We observed that some of these peptides (n=79) were predicted to have MHC Class I binding epitope regions associated with at least one of the Class I HLA alleles with IC50 < 500nm (Supplementary Table S10). These antigenic peptides were predicted to bind with 20 most frequently occurring HLA Class I alleles, in which HLA*02:03 and HLA*02:06 were predicted to present the highest number of epitope residues. To further investigate if these peptides are specific to the coronavirus family, we performed the BLAST comparison with 2807 known viral antigenic peptides of bacteria, influenza-and corona-virus family (non-SARS CoV-2) from IEDB (with at least one T cell assay evidence) and filtered out matching peptides (Blast e-value < 1 & identity > 70%). Finally, we present 48 high-affinity HLA-binding peptides which are unique to the SARS-CoV-2 proteome, not common to bacteria, influenza and corona virus family antigenic peptides within a given threshold range (Supplementary Table S11) with 14 high confidence HLA Class I binding peptides with IC50 < 50nm (Table 3).

## Discussion

We have applied two independent, complementary and systematic bioinformatic approaches in order to identify potentially cross-reactive allergen- and SARS-CoV-2-T cell epitopes. Our *in silico* analysis revealed numerous candidate epitope pairs, including previously published and predicted peptides, while both applied pipelines highlighted an important role of MHC class I inhalant allergens. Although the frequency of allergen-specific CD8^+^ T cells is likely to be low, rare cell subsets have been quite often shown to play an important pathophysiological role^27^, and new technologies and bioinformatic approaches for identification of such populations are steadily emerging^28^. Quite importantly, the SARS-CoV-2 Nsp6141–149, which was identified among our top potentially cross-reactive epitope pairs, has been recently described by an independent group.^29^ To our knowledge, this is the first report on *in silico* predicted T cell epitope cross-reactivity between SARS-CoV-2 and allergens. While a limitation of our study is the *in silico* nature of the work, the sequence homology between SARS-CoV-2 and clinically relevant respiratory allergens is along the lines of previously reported cross-reactivity between RNA virus- and allergen-derived peptides at the level of T memory cells.^4^ Moreover, our current findings generate further hypotheses in how the adaptive immune system responds differentially with respect to the atopy status of the host. Our present study warrants an immediate investigation of these predicted T cell epitopes to link their possible role in driving the immune response against the SARS-CoV-2 and eventually shape COVID-19 outcome.

There are several different avenues through which the similarities may influence the host immune response. For instance, in hosts sensitized to one of the predicted aeroallergens, the identified similarities with the SARS-CoV-2 proteome may be protective if they prevent an overwhelming Th1 response and the accompanying cytokine storm. Furthermore, allergen-specific T cells may develop a memory response against heterologous SARS-CoV-2 epitopes, which is faster and more efficient. Conversely, such heterologous immune responses could have an adverse outcome by attenuating the antiviral response. T2 immune bias could potentially lead to inadequate virus clearance due to attenuated CD8^+^ responses. Indeed, there is evidence of a reciprocal relationship between atopy and production of type I and III Interferons in response to viral infections^30^. Given that underlying atopic conditions have not been identified as a significant risk factor for severe clinical courses in those infected with SARS-CoV-2, the epitope homology most likely plays a protective role^7,8^. Interestingly, Jackson et al recently reported that nasal epithelial cells from children with atopic asthma express significantly lower levels of ACE2 receptor as compared to cells from children without asthma or with non-atopic asthma^31^. Similarly, another study using adult bronchial brush samples showed an inverse correlation between ACE2 gene expression and a Th2 dependent gene expression signature^32^. Differential expression of ACE2 receptors among atopic individuals could represent a distinct and unrelated mechanism of action in this context. Our *in silico* data provide ground to investigate the role of cellular immune responses in regards to the interaction between atopy/asthma and COVID-19. Indeed, the role of SARS-CoV-2-specific T cells in exposed and non-exposed individuals, thereby underlining the importance of heterologous immunity, has been very recently described^33,34^. Further experimental studies are needed to explore the involved pathogenetic mechanisms and potential clinical implications of underlying aeroallergen sensitization on the immune response to SARS-CoV-2.

## Supplementary Material

Supplement

Supplement

Supplement

Supplement

Supplement

Supplement

Supplement

Supplement

## Figures and Tables

**Table 1.** The Top 5 candidate human HLA class I T cell potentially cross-reactive epitope pairs between SARS-CoV-2 and aero-and food-allergens based on pair combined score and application of additional clinical and conservation related criteria (see Suppl. Figure 1) (pipeline 1). Pcs=pair combined score

MHC I		Top 30 pair combined score					
		candidate	allergen epitope	protein family	viral epitope	MHC allele	pcs
**AERO**	Nr.1	Mus m 1	GSNTFTILK	Lipocalin	TSNSFDVLK	HLA-A_11_01	8188
	Nr. 2	Asp f 5	MLYEVLWNL	Fungalysin metalloprotease	YLYALVYFL	HLA-A_02_01	5974
	Nr. 3	Aln g 1	SGVSPVSYQK	Bet v 1 family	ATSRTLSYYK	HLA-A_11_01	2413
	Nr.4	Phl p 5	KYKTFVATF	Group 5/6 grass pollen allergen	MFDAYVNTF	HLA-A_24_02	1910
	Nr. 5	Mus m 1	GSNTFTILK	Lipocalin	VTNNTFTLK	HLA-A_11_01	3286

**FOOD**	Nr. 1	Gal d 5	FLGHFIYSV	Serum albumin	TMADLVYAL	HLA-A_02_01	1408
	Nr. 2	Gal d 6	YLLDLLPAA	Lipoprotein	TLMNVLTLV	HLA-A_02_01	4185
	Nr. 3	Gal d 6	RPAYRRYLL	Lipoprotein	RPPLNRNYV	HLA-B_07_02	2274
	Nr. 4	Cor a 1	APHGGGSIL	Bet v 1 family	VPGLPGTIL	HLA-B_07_02	2571
	Nr. 5	Gal d 6	KVFRFSMFK	Lipoprotein	LVASIKNFK	HLA-A_11_01	1194

**Table 2.** The Top 5 candidate human HLA class II T cell potentially cross-reactive epitope pairs between SARS-CoV-2 and aero-and food-allergens based on pair combined score and application of additional clinical and conservation related criteria (see Fig.1) (pipeline 1) Pcs=pair combined score

MHC II		Top 30 pair combined score					
		candidate	allergen epitope	protein family	viral epitope	MHC allele	pcs
**AERO**	Nr.1	Phl p 5	FVATFGAAS	Group 5/6 grass pollen allergen	FSSTFNVPM	HLA-DRB1_04_01	87
	Nr. 2	Asp f 4	LTALAAGSA	Unclassified	VTALRANSA	HLA-DRB1_01_01	479
	Nr. 3	Phl p 5	FVATFGAAS	Group 5/6 grass pollen allergen	FSSTFNVPM	HLA-DRB1_04_01	53
	Nr.4	Phl p 5	FVATFGPAS	Group 5/6 grass pollen allergen	FSSTFNVPM	HLA-DRB1_04_01	29
	Nr. 5	Phl p 5	FKVAATAAN	Group 5/6 grass pollen allergen	FSSTFNVPM	HLA-DRB1_04_01	38

**FOOD**	Nr. 1	Gal d 5	FLYAPAILS	Serum albumin	FYILPSIIS	HLA-DRB1_01_01	299
	Nr. 2	Gal d 6	ILVDAVLKE	Lipoprotein	VVADAVIKT	HLA-DRB1_03_01	113
	Nr. 3	Gal d 6	VYSDVPIEK	Lipoprotein	VVADAVIKT	HLA-DRB1_03_01	29
	Nr. 4	Ara h 1	FIMPAAHPV	Cupin	FVMMSAPPA	HLA-DRB1_01_01	258
	Nr. 5	Gal d 5	FLYAPAILS	Serum albumin	FLYENAFLP	HLA-DRB1_01_01	81

**Table 3.** HLA-I binding high confidence (IC50 < 50nm) SARS-CoV-2 antigenic peptides (pipeline 2)

Allele	HLA-I-Binding Peptide	IC50	SARS-CoV-2 Protein name
HLA-A*68:01	NIFGTVYEK	6	R1AB_SARS2_Replicase_polyprotein
HLA-A*02:06	YTVELGTEV	9.4	R1A_SARS2_Replicase_polyprotein
HLA-A*68:02	YTVELGTEV	10.8	R1A_SARS2_Replicase_polyprotein
HLA-B*15:03	LASHMYCSF	10.8	R1A_SARS2_Replicase_polyprotein
HLA-B*40:02	HEGKTFYVL	11	SPIKE_SARS2_Spike_glycoprotein
HLA-B*40:01	GETLPTEVL	11.9	R1AB_SARS2_Replicase_polyprotein
HLA-A*02:06	TVYEKLKPV	13.4	R1AB_SARS2_Replicase_polyprotein
HLA-A*30:02	ASHMYCSFY	13.9	R1A_SARS2_Replicase_polyprotein
HLA-B*40:01	HEGKTFYVL	13.9	SPIKE_SARS2_Spike_glycoprotein
HLA-A*11:01	NIFGTVYEK	24	R1AB_SARS2_Replicase_polyprotein
HLA-B*35:01	LASHMYCSF	24.5	R1A_SARS2_Replicase_polyprotein
HLA-A*68:02	TVYEKLKPV	26	R1AB_SARS2_Replicase_polyprotein
HLA-A*02:01	WLTNIFGTV	34.1	R1AB_SARS2_Replicase_polyprotein
HLA-A*02:06	WLTNIFGTV	34.7	R1AB_SARS2_Replicase_polyprotein
HLA-B*15:03	LTNIFGTVY	35.7	R1AB_SARS2_Replicase_polyprotein
HLA-B*15:25	LASHMYCSF	39.1	R1A_SARS2_Replicase_polyprotein
HLA-B*15:25	LTNIFGTVY	39.8	R1AB_SARS2_Replicase_polyprotein
HLA-A*02:01	TVYEKLKPV	47.8	R1AB_SARS2_Replicase_polyprotein
HLA-B*15:01	LASHMYCSF	48.1	R1A_SARS2_Replicase_polyprotein
HLA-B*15:03	ASHMYCSFY	49	R1A_SARS2_Replicase_polyprotein

## LIST OF ABBREVIATIONS

ACE2: Angiotensin Converting Enzyme 2
ARDS: Acute Respiratory Distress Syndrome
BLAST: Basic Local Alignment Search Tool
HLA: Human Leukocyte Antigen
IEDB: Immune Epitope Database
MHC: Major Histocompatibility Complex
NCBI: National Center for Biotechnology Information
RNA: ribonucleic acid
+ssRNA: positive-sense single-stranded RNA
Th 1: t-helper 1
Th 2: t-helper 2
