# Supplementary material for: Homologies between SARS-CoV-2 and allergen proteins may direct T cell-mediated heterologous immune responses
Source: Res Sq. 2020 Oct 6:rs.3.rs-86873. Preprint. [Version 1] doi: 10.21203/rs.3.rs-86873/v1 (PMC7553154; doi:10.21203/rs.3.rs-86873/v1)
Supplement: Supplement [file d93f235026cb4bb5153fadf8.pdf]

Supplementary Information

Homologies between SARS-CoV-2 and allergen proteins may direct T cell-mediated heterologous immune responses

Kathrin Balz[1*], MSc, Meng Chen[2*], MD, Abhinav Kaushik[2*], PhD, Franz Cemic[3], PhD, Vanessa Heger[3], BSc, Harald Renz[1], MD, Kari Nadeau[2#], MD, PhD, Chrysanthi Skevaki[1#], MD

[1] Institute of Laboratory Medicine, Universities of Giessen and Marburg Lung Center (UGMLC), Philipps University Marburg, German Center for Lung Research (DZL), Marburg, Germany

[2] Sean N. Parker Center for Allergy and Asthma Research at Stanford University and Division of Pulmonary, Allergy & Critical Care Medicine, Stanford, CA, USA.

[3] TH Mittelhessen, Department of Computer Science, University of Applied Sciences Gießen, Hessen, Deutschland

[*] equal contribution

[#] equal contribution

MATERIALS AND CORRESPONDENCE

Chrysanthi Skevaki, PD Dr.

Institute of Laboratory Medicine, Philipps University Marburg

Baldingerstrasse, 35043 Marburg, Germany

Phone +49 6421 586 6235

Email: chrysanthi.skevaki@uk-gm.de

Supplementary Figures

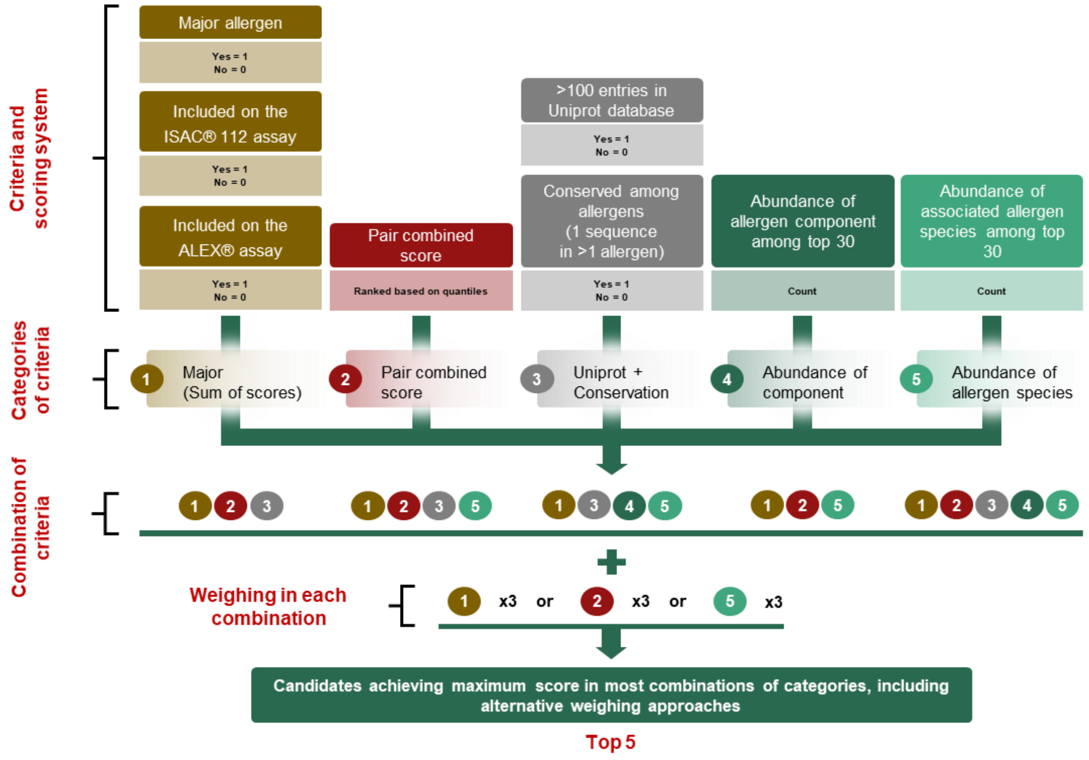

Supplementary Figure S1. Criteria and workflow for further scoring of the top 30 potentially cross-reactive predicted allergen-derived T cell epitopes for pipeline 1. Individual molecular allergen components or their associated peptides among the top 30 potentially cross-reactive epitope pairs were scored based on (1) whether they evoked IgE production in >50 % of patients with associated clinical allergy as per WHO/IUIS Allergen Nomenclature Sub-committee (http://allergen.org/index.php) and whether they are included in the commercially available ImmunoCAP ISAC®112 or ALEX® diagnostics assays; (2) pair combined score, defined in 4 ranges, with the help of percentiles : ≤ 10 % = 0; [20-50 %] = 1; [50-90 %] = 2; [90-100 %] = 3; (3) whether they achieve more than 100 entries when entering the epitope sequence in Uniprot and whether the specific epitope sequence is also contained in proteins of other allergen sources and (4) how abundant the specific allergen component or (5) associated allergen source is within the top 30 candidates. Five different combinations of the aforementioned categories were defined and scored, and the cumulative score was calculated for each allergen epitope. Additionally, the cumulative score was calculated three more times, each time multiplying another category by a factor of 3 in order to critically compare alternative weighing of the associated criteria. Allergens were ultimately prioritized for further testing based on the frequency of achieving the maximum score in each of the separately weighed scoring approaches as described above.

Supplementary Tables

Supplementary Table S3: The most prevalent human HLA alleles, which were targeted for the in silico epitope prediction in pipeline 1

Human MHC Class I[1]	Human MHC Class II[2]
HLA-A*01:01	DRB1*01:01
HLA-A*02:01	DRB1*03:01
HLA-A*11:01	DRB1*04:01
HLA-A*24:02	
HLA-B*07:02	
HLA-B*40:02	

1=epitopes with a length of 9 or 10 amino acids

2=epitopes with a length of 15 amino acids

Supplementary Methods

Calculation of the pair combined score:

The pair combined score takes the binding affinity of predicted viral and allergen epitopes to MHC molecules into consideration, as well as the score from the pairwise alignment and cross-entropy (cut-off 0,8): Pair combined score= 1/binding affinity (nM) (Virus) * 1/binding affinity (nM) (Allergen) * score PwA. Therefore, a higher score is associated with an increasing probability for MHC binding and a higher degree of similarity between the virus and allergen epitope.

Scoring system:

A scoring system was developed and five categories of criteria were formed, summing up the scores of individual criteria in one group (Figure S1). As a next step, five different combinations of the aforementioned categories were defined and the cumulative score was calculated for each allergen epitope. Additionally, the cumulative score was calculated three more times, each time multiplying another category by a factor of 3 in order to critically compare alternative weighing of the associated criteria. The new Top 5 allergens and associated virus epitopes were subsequently ranked based on the frequency of achieving the maximum score in each of the separately weighed scoring approaches as described above.
